# Q61 mutant-mediated dynamics changes of the GTP-KRAS complex probed by Gaussian accelerated molecular dynamics and free energy landscapes†

**DOI:** 10.1039/d1ra07936k

**Published:** 2022-01-11

**Authors:** Jianzhong Chen, Qingkai Zeng, Wei Wang, Qingquan Hu, Huayin Bao

**Affiliations:** School of Science, Shandong Jiaotong University Jinan 250357 China chenjianzhong1970@163.com jzchen@sdjtu.edu.cn; School of Pharmacy, Shandong University of Traditional Chinese Medicine Jinan 250355 China baohuayin@163.com

## Abstract

Understanding the molecular mechanism of the GTP-KRAS binding is significant for improving the target roles of KRAS in cancer treatment. In this work, multiple replica Gaussian accelerated molecular dynamics (MR-GaMD) simulations were applied to decode the effect of Q61A, Q61H and Q61L on the activity of KRAS. Dynamics analyses based on MR-GaMD trajectory reveal that motion modes and dynamics behavior of the switch domain in KRAS are heavily affected by the three Q61 mutants. Information of free energy landscapes (FELs) shows that Q61A, Q61H and Q61L induce structural disorder of the switch domain and disturb the activity of KRAS. Analysis of the interaction network uncovers that the decrease in the stability of hydrogen bonding interactions (HBIs) of GTP with residues V29 and D30 induced by Q61A, Q61H and Q61L is responsible for the structural disorder of the switch-I and that in the occupancy of the hydrogen bond between GTP and residue G60 leads to the structural disorder of the switch-II. Thus, the high disorder of the switch domain caused by three current Q61 mutants produces a significant effect on binding of KRAS to its effectors. This work is expected to provide useful information for further understanding function and target roles of KRAS in anti-cancer drug development.

## Abstract 2

Q61 mutants induce structural disorder of the switch domain in KRAS and affect binding of KRAS to effectors.

## Graphical abstract

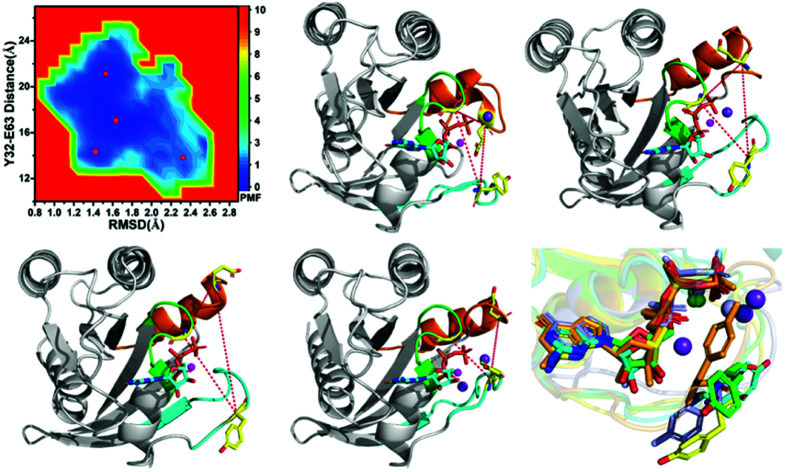

## 1 Introduction

RAS proteins HRAS, KRAS and NRAS are considered as significant family members of small guanosine triphosphatases (GTPases) and they can be functionally utilized as a molecular switch to regulate cell growth and differentiation in intracellular signaling pathways.^1,2^ The GTPase activating proteins (GAPs) accelerate the hydrolysis reaction of guanosine triohosphte (GTP) into guanosine diphosphate (GDP), which leads to an inactive state of RAS proteins.^3^ Guanosine exchange factors (GEFs) catalyze the transformation of GDP toward GTP and induce an active state of the GTP-bound RAS proteins.^3^ Multiple signaling pathways playing a key role in cell proliferation and survival can be activated through interactions of GTP-bound RAS proteins with effectors, namely RAF, phosphoinositide 3-kinase (PI3K) and Ral guanine nucleotide dissociation stimulator (RalGDS).^4^ The previous reports suggested that the active RAS proteins mediated by residue mutations promote tumorigenesis through hyperactivating the downstream signal pathways.^4–8^ Among mutations of three RAS isoforms, KRAS oncogenes account for ∼82% of RAS mutations relating with human cancer based on information from COSMIC.^9^ Therefore, KRAS has been an interesting focus on design of clinically available drugs toward human cancer treatment.

**Fig. 1:**
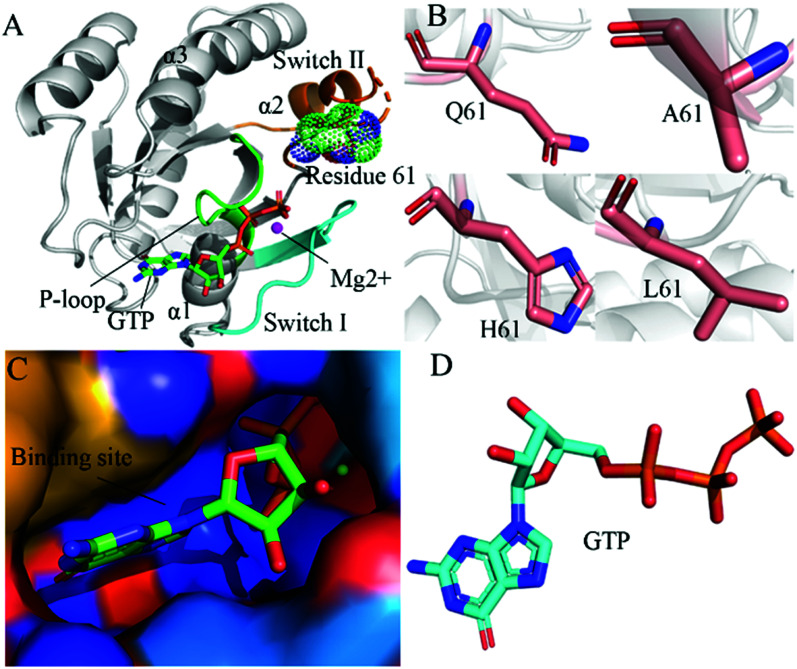
Molecular structures: (A) the GTP-bound KRAS shown by using the crystal structure (PDB ID: 6MNX), in which KRAS, GTP, mutated site and magnesium ion (Mg^2+^) are displayed in cartoon, stick, dot and ball modes, respectively, moreover the P-loop, switch-I and switch-II exhibited in green, cyan and orange, (B) Q61 and mutants A61, H61 and L61, (C) binding pocket of GTP depicted in surface styles and (D) GTP shown in stick patterns.

By now, a number of molecular structures of GTP- and GDP-bound KRAS have been determined by X-ray and NMR experiments from different work groups,^10–15^ which provide significant structural basis for understanding function of KRAS. According to function of structural domains, the conserved catalytic domain of KRAS is divided into two functional lobes, namely the effector lobe (residues 1–86) and the allosteric lobe (residues 87–166). The effector lobe is located at the N-terminal region of the catalytic domain, including P-loop (residue 10–17), switch-I (residues 30–40) and switch-II (residues 60–76), and these secondary structures form the main binding sites (Fig. 1A).^16^ The allosteric lobe is involved in a α-helical dimerization interface and binding motifs of GTP or GDP's nucleotide base and functions as a relaying of information through the protein.^16–18^ In the above mentioned structures, the switch-I and switch-II generate functional interactions with GEFs and GAPs or effectors of KRAS, indicating that the conformational transformation between switch-I and switch-II highly affects the activity of KRAS.^19–24^ Hence it is of high significance to deeply explore molecular mechanism underlying conformational changes of KRAS for understanding how to tune the activity of KRAS.

The previous works suggested that mutations significantly affect the conformational alterations of the switch-I and switch-II in KRAS and change binding activity of KRAS to its regulators and effectors.^25–33^ From residue substitutions at different sites, G12, G13 and Q61 mutations respectively account for 89%, 9% and 1% of the observed mutants of KRAS.^34^ In addition, the effect of mutations at the other sites on the GTP- and GDP-KRAS binding were also investigated by different work groups.^29,35–37^ Based on significant roles of the P-loop in function of KRAS, many experimental and theoretical works have focused on insights into impacts of G12 and G13 mutations on the conformational changes of KRAS and the results provide molecular mechanisms underlying the regulation of mutations from these two sites on the activity of KRAS.^38–50^ Although the number of works correlating with Q61 mutations in KRAS is less than that of G12 and G13 mutations, Q61 mutations also play a vital role in regulations on binding activity of KRAS to its effectors and regulators. For instance, the X-ray crystal structure of the GTP-associated Q61H determined by Zhou *et al.* unveiled that a hyperdynamic switch-II induces a more stable interaction with switch-I and verified that the enhanced RAF activity stems from not only the absent intrinsic GTP hydrolysis activity but also the improved affinity for RAF.^51^ The investigation from Yan *et al.* suggested that the GMPPNP-bound Q61L KRAS generates a stronger binding to NF1 than the GMPPNP-bound Q61R one and provided a new mechanistic insight about how SPRED1 interacts with neurofibromin.^52^ The work of Buhrman *et al.* on transformation efficiency of Q61 mutations clarified a molecular mechanism of intrinsic hydrolysis in the RAF/RAS complex and elucidated structural features in different Q61 mutants correlating with their potency of cell transformation.^53^ Despite these successful studies, dynamics information involved in effect of Q61 mutations on the activity of KRAS is insufficient. Therefore, it is highly requisite to further explore Q61 mutant-mediated conformational changes of KRAS for deeply understanding function of KRAS.

Up to now, multiple simulation methods, including conventional molecular dynamics (cMD),^54–62^ replica-exchange molecular dynamics (REMD),^63,64^ accelerated molecular dynamics (aMD)^65–69^ and Gaussian accelerated molecular dynamics (GaMD)^70–78^*etc.*, have been proposed to probe conformational changes of receptors because of ligand bindings and residue mutations. Compared to cMD, MR-GaMD simulations can obtain more rational conformational sampling on receptors.^71,76^ Recently, MD simulations were also applied to successfully investigate impacts of Q61 mutations on the activity of KRAS. The NMR S^2^ calculated by Kumar *et al.* with the DynaMine suggested that Q61H interferes with water molecule coordination and affects hydrolysis of GTP.^24^ The results from MD simulations of Vatansever *et al.* indicated that Q61H on the switch-II does not generate obvious influences on the fluctuations of the switch-II in active KRAS but evidently affects the flexibility of inactive protein.^40^ Lu *et al.* performed MD simulations on KRAS of multiple mutations and their studies verified that Q61H is more prone to alter the KRAS4B–GTP conformation to the active state than the G12C mutation.^34^ Mutations at Q61 play vital roles in regulations on bindings of KRAS to the effectors and regulators. Thus, it is still high significance to further explore dynamics changes of KRAS by using rational conformational sampling technology for efficiently understanding the role of KRAS in drug targets toward cancer treatments.

In this work, three mutations Q61A, Q61H and Q61L at the GTP-bound KRAS, observed at the experimental works,^10,53,79^ were selected to decipher influences of Q61 mutants on binding activity of KRAS. Q61 is a residue without charge and located at the switch-II. The sidechain of Q61 is replaced by a small hydrophobic alkyl, a big hydrophobic alkyl and a hydrophobic ring in Q61A, Q61L and Q61H, respectively (Fig. 1B). Binding pocket and molecular structure of GTP were separately displayed in Fig. 1C and D. Insights into impacts of the changes in sizes of hydrophobic sidechains of Q61 mutants on conformational alterations of KRAS are helpful for understanding molecular mechanism for tuning binding activity of KRAS. To reach our current aims, multiple replica GaMD (MR-GaMD) simulations were employed to enhance conformational sampling of KRAS. Principal component analysis (PCA), construction of free energy landscapes (FELs) and interaction network analysis were performed to explore Q61 mutant-mediated regulation on conformational changes of KRAS. This work is also expected to provide useful information for deeply understanding activity and function of KRAS.

## 2 Methods

### 2.1 Modeling simulated systems

The crystal structure 6MNX was extracted from protein data bank (PDB) to obtain the initial structure of the GTP-bound Q61H KRAS.^51^ Two missing residues Y64 and S65 in the A section of 6MNX were repaired with the program MODELLER.^80^ To keep atomic coordinate consistency, residue H61 in the repaired structure was separately changed into Q61, A61 and L61 to obtain the initial structures of the GTP-bound WT, Q61A and Q61L KRAS. A Web server H++ 3.0 (http://biophysics.cs.vt.edu/H++) was used to check and assign rational protonated states to residues of KRAS.^81^ All crystal water molecules and magnesium ion (Mg^2+^) were left at the initial structures of four simulated systems. The Leap module in Amber 20 (ref. 82 and 83) was utilized to obtain force field parameters of four simulated systems based on the following procedure: (1) forming the chemical bonds between heavy atoms and hydrogen atoms at the crystal structures, (2) producing force field parameters of GTP by aid of the work of Meagher *et al.*,^84^ (3) generating force field parameters of the WT and mutated KRAS with *ff*14SB force field,^85^ (4) building an octahedral water box of 12 Å buffer with the TIP3P model^86^ to solve the GTP-KRAS complexes and assigning force field parameters to water molecules and (5) neutralizing the simulated systems with the appropriate sodium ions (Na^+^) in a salt environment of 0.15 M NaCl and yielding force field parameters of Na^+^ and Mg^2+^ by using the Aqvist force field.^87^

### 2.2 MR-GaMD simulation

The program pmemd.cuda inlayed at Amber 20 was employed to carry out all the following simulations.^88^ 3000-step steepest descent minimization, 5000-step conjugate gradient minimization, 2 ns heating process of 0 to 310 K at the *NVT* condition and 2 ns equilibrium process of 310 K at the *NPT* condition were sequentially run to deeply relax every simulated system. Then, the relaxed structure was utilized to execute 200 ns cMD simulation on each simulated system. Two structures randomly extracted from the previous cMD simulation were used as initial conformation to restart two new 200 ns cMD simulations. Three ending structures from the above cMD simulations were adopted to perform three independent MR-GaMD simulations and each simulation was run for 1.6 μs.

GaMD simulations can efficiently leap over energy barrier of simulated systems and enhance conformational samplings with the following potential function described by eqn (1) and (2)1
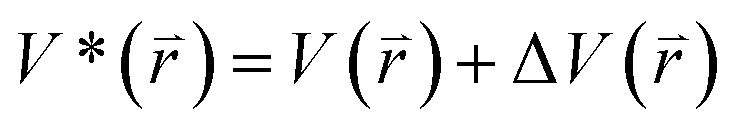
2
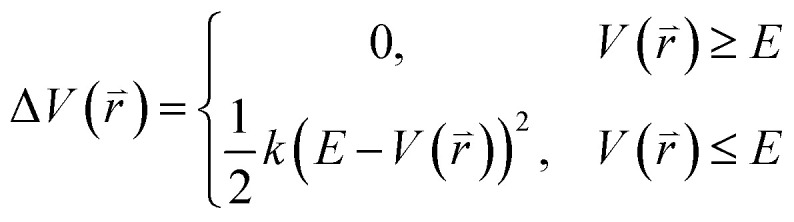
where 
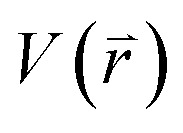
 represents potential function of simulated systems and 
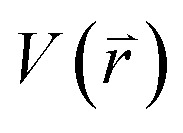
 is revised as 
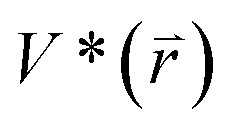
 when 
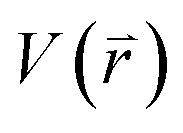
 is lower than a threshold energy *E*. Two parameters *E* and *k* can be updated based on the following principals3
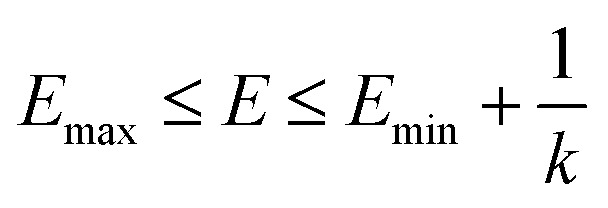
4
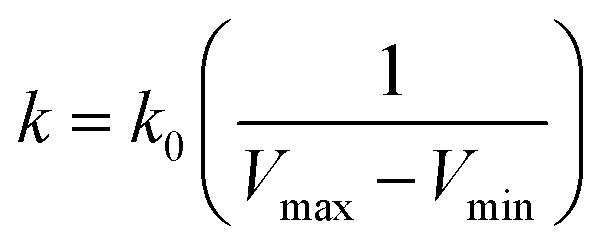
in which when *E* is lower than an energy *E* = *E*_max_, *k*_0_ is obtained from the eqn (5) as below5
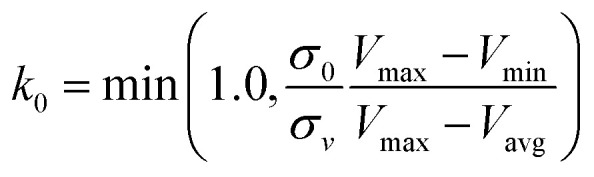
but when *E* is set as an upper bound 
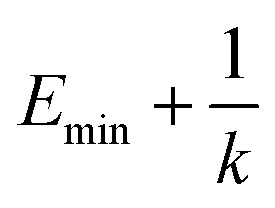
, *k*_0_ is got from the eqn (6)6
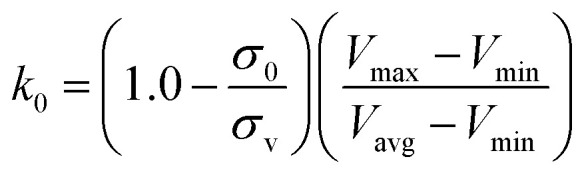
where *V*_max_, *V*_min_ and *V*_avg_ correspond to the minimum, maximum, and averaged potential energy of each system, respectively. The parameter *σ* represents standard deviation of potential energies and the parameter *σ*_0_ is artificially set as an upper limit to accurately reweight the original energy profile of simulated systems. In our work, 4.8 μs MR-GaMD simulations, composed of three-replica GaMD simulations of 1.6 μs, were run on the GTP-bound WT, Q61A, Q61H and Q61L KRAS to rationally sample conformations of KRAS. Three-replica GaMD trajectories were connected into a single integrated MR-GaMD trajectory to facilitate the post-processing analysis. A program PyReweighting^89^ provided by Miao was employed to decode energy profile of KRAS and probe conformational changes of KRAS. Through all simulations involved in this work, chemical bonds linking hydrogen with heavy atoms were restrained by utilizing the SHAKE algorithm.^90^ The Langevin dynamics,^91^ assigned as a collision frequency of 2.0 ps^−1^, was used for regulation on temperatures of four simulated systems. The particle mesh Ewald (PME) was wielded to estimate electrostatic interactions between atoms and the cutoff value for treating non-bonded interactions was set as 12 Å.

### 2.3 Principal component analysis

To recognize correlated motions relating with functional significance, PCA was used in our current work.^92–95^ The first step of PCA is to build a covariance matrix *C* by using eqn (7)

7 *C* = 〈(*q*_*i*_−〈*q*_*i*_〉)(*q*_*j*_−〈*q*_*j*_〉)^*T*^〉

where *q*_*i*_ and *q*_*j*_ are the Cartesian coordinates of the *i*th and *j*th *C*_α_ atoms in KRAS while 〈*q*_*i*_〉 and 〈*q*_*j*_〉 indicate their averaged positions. The average is estimated by superimposing the single integrated MR-GaMD trajectory with a defined reference structure to remove overall translations and rotations with a least-square fit procedure.^96^ The second step of PCA is to diagonalize the symmetric matrix *C* into a diagonal one *A* with an orthogonal coordinate transformation matrix *T* based on the following equation

8 *A* = *T*^*T*^*C*_*ij*_*T*

from which the diagonal elements represent the eigenvalues *λ*_*i*_ and the columns signify the eigenvectors characterizing the motion direction of movement relative to 〈*q*_*i*_〉. The third step of PCA is to describe a single correlated displacement of a group of atoms in a multidimensional space using the eigenvector and explain the amplitude of the motion along an eigenvector, which can rationally reflect conformational changes of the domain in KRAS. In this work, PCA was carried out by utilizing the CPPTRAJ module in Amber 20.^97^ The software VMD^98^ was employed to visualize the data from PCA, plot pictures and unveil impacts of Q61 mutants on conformational alterations of KRAS.

### 2.4 Cross-correlation analysis

To check the extent of correlated movements of structural domain in KRAS, cross-correlation matrix, *C*_*ij*_, was calculated by using the *x*, *y* and *z* coordinates of the *C*_α_ atoms in KRAS based on the following equation^99^

9 *C*_*ij*_=(Δ***r***_*i*_ × Δ***r***_*j*_)/(〈Δ***r***_*i*_^2^〉〈Δ***r***_*j*_^2^〉)^2^

where Δ***r***_*i*_ and Δ***r***_*j*_ are the displacement vectors of atoms *i* and *j* relative to their averaged positions while the angle brackets indicate ensemble averages over the structures recorded at the single integrated MR-GaMD trajectory. The element values of cross-correlation matrix are located at a range from −1 to 1. The *C*_*ij*_ of positive values describe the positively correlated motions between atoms *i* and *j* while the *C*_*ij*_ of negative values reflect the anti-correlated movements of atoms *i* relative to *j*. The color-coded patterns were adopted to characterize the extent of correlated motions. In our current study, the module CPPTRAJ in Amber 20 was wielded to execute cross-correlation analysis.

### 2.5 Analysis of interaction network

Identification of interaction network plays an important role in probing ligand-receptor binding mechanisms. Hydrogen bonding interactions (HBIs) and hydrophobic interactions are two favorable factors for bindings of ligands to receptors. In this work, the module CPPTRAJ inlayed in Amber 20 was applied to analyze HBIs between GTP and KRAS. A protein−ligand interaction profiler (PLIP) server^100^ was employed to recognize interaction networks and types of GTP with KRAS. Meanwhile the software PyMOL (https://www.pymol.org.) was utilized to depict hot interaction spots of GTP with KRAS.

## 3 Results and discussion

### 3.1 Structural fluctuations and flexibilities of KRAS

**Fig. 2:**
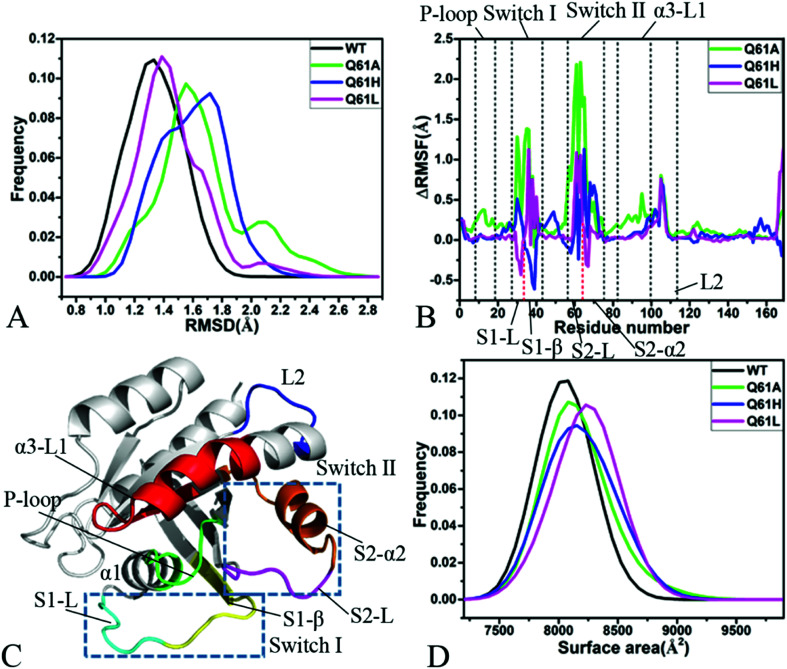
Structural fluctuations and flexibilities: (A) frequency distributions of backbone RMSDs, (B) RMSF difference of *C*_α_ atoms between the WT and mutated KRAS, (C) structural domains corresponding to obvious changes of RMSF values and (D) frequency distributions of molecular surface areas.

Root-mean-square deviations (RMSDs) were computed by using the coordinates of backbone atoms from KRAS recorded at the single integrated MR-GaMD trajectory to understand changes in structural fluctuations of KRAS due to Q61 mutations. Frequency distributions of RMSDs were plotted in Fig. 2A. RMSDs of the GTP-bound Q61A, Q61H and Q61L KRAS are increased by 0.22, 0.39 and 0.10 Å compared to that of the GTP-bound WT KRAS, respectively, moreover the distribution shape of RMSDs of three mutated KRAS moves toward the right. Therefore, three mutations Q61A, Q61H and Q61L strengthen structural fluctuations of KRAS relative to the WT one and the changes in structural stability certainly affect interactions of KRAS with GEFs and GAPs or effectors.

Difference in root-mean-square fluctuations (RMSFs) of the *C*_α_ atoms between the mutated and WT KRAS was calculated by using the equation ΔRMSF = *RMSF*_mutated_ − *RMSF*_WT_ to explore impacts of Q61 mutants on structural flexibility of KRAS (Fig. 2B). As a result, Q61A, Q61H and Q61L yield obvious effect on the switch-I and switch-II of KRAS. According to Fig. 2C, the switch-I consists of two parts (S1-L and S1-β) while the-switch II is composed of two parts (S2-L and S2-α2). Q61A evidently enhances the structural flexibility of the switch-I relative to the WT KRAS. By comparison with the WT KRAS, Q61H increases the structural flexibility of S1-L in the switch-I but decreases that of S1-β, on the contrary, Q61L weakens the flexibility of S1-L but strengthens that of S1-β (Fig. 2B and C). Similar to the switch-I, Q61A highly strengthens the flexibility of the switch-II relative to the WT KRAS. Compared to the WT KRAS, Q61H slightly reduces the flexibility of S2-L in the switch-II but evidently enhances that of S2-α2, differently, Q61L strengthens the flexibility of S2-L but softly weakens that of S2-α2 (Fig. 2B and C). Meanwhile, Q61A, Q61H and Q61L increase the structural flexibility of a loop L2 linking α3 with a β-strand relative to the WT KRAS. In addition, Q61A evidently enhances the structural flexibility of P-loop and α3-L1 compared to the WT KRAS. According to structural information, the switch-I and switch-II are involved in functional interactions of KRAS with GEFs and GAPs or effectors,^21^ thus the alterations in the flexibility of these two switches necessarily affect the activity of KRAS, which is also supported by the previous works.^34,40^

Molecular surface areas (MSAs) of KRAS were also computed to probe influences of Q61 mutants on total flexibility of KRAS and frequency distribution of MSAs was displayed in Fig. 2D. The MSAs of the GTP-bound Q61A, Q61H and Q61L KRAS are increased by ∼72, 150 and 221 Å^2^ by comparison with that of the WT KRAS, respectively, moreover the distribution shape of MSAs in three mutated KRAS move toward the right. Hence it is concluded that Q61A, Q61H and Q61L enhance the total flexibility of KRAS relative to that of the WT KRAS.

Based on the aforementioned information, Q61A, Q61H and Q61L obviously increase the structural fluctuation of KRAS by referencing the WT state. Meanwhile, three mutants Q61A, Q61H and Q61L not only evidently alter the local flexibility of the switch-I, switch-II and loop L2 in KRAS but also strengthen the total flexibility of KRAS. These structural changes inevitably generate significant influences on binding of KRAS to GEFs and GAPs or effectors.

### 3.2 Changes in dynamics behavior of KRAS affected by Q61 mutations

Cross-correlation analysis was executed to uncover effect of Q61 mutations on correlated motions between residues of KRAS and the results were visually display in ESI Fig. S1.† Color-coded styles were adopted to reflect the extent of correlated motions. It is observed that Q61A, Q61H and Q61L produce different influences on correlated movements of KRAS. In the WT state, the switch-II yields obvious anticorrelated motions (blue) relative to the P-loop but the switch-I hardly produces correlated movements relative to the P-loop (Fig. S1A†). In the meantime, a slight anticorrelated motion (light blue) also appear between the switch-II and switch-I. In addition, the structure α3-L1 not only generates a strong positive correlated motion (yellow) relative to the P-loop but also produces a weak anticorrelated movement relative to the switch-II (Fig. S1A† and 2C).

By comparison with the WT KRAS, Q61A enhances the anticorrelated motion between the switch-II and the P-loop but Q61H and Q61L highly weaken this anticorrelated motion (Fig. S1B–D†). Compared to the WT state of KRAS, Q61A obviously strengthens the anticorrelated motions (dark blue) of the switch-I relative to the P-loop and the α3-L1 relative to the switch-II, however Q61H and Q61L hardly affect these two anticorrelated motions. Besides, Q61A extremely enhances the positive correlated movement (yellow and red) between the α3-L1 and the P-loop, but Q61H and Q61L do not bring obvious effect on this positive correlated motion (Fig. S1B–D† and 2C).

In general, eigenvalues obtained from PCA are utilized to reflect total motion intensity of proteins. For this study, function of eigenvalues *vs.* eigenvector indexes were depicted in Fig. S2.† In fact, the first several bigger eigenvalues describe mainly concerted motions of structural domain in KRAS. As shown in Fig. S2,† the first five eigenvalues account for 60.1, 86.5, 82.8 and 83.3% of the total movements of the GTP-associated WT, Q61A, Q61H and Q61L KRAS, respectively. By referencing the WT KRAS, the first eigenvalue of three mutated KRAS is extremely improved compared to that of the WT KRAS. Thus Q61A, Q61H and Q61L highly enhance the total motion intensity of KRAS relative to the WT one.

**Fig. 3:**
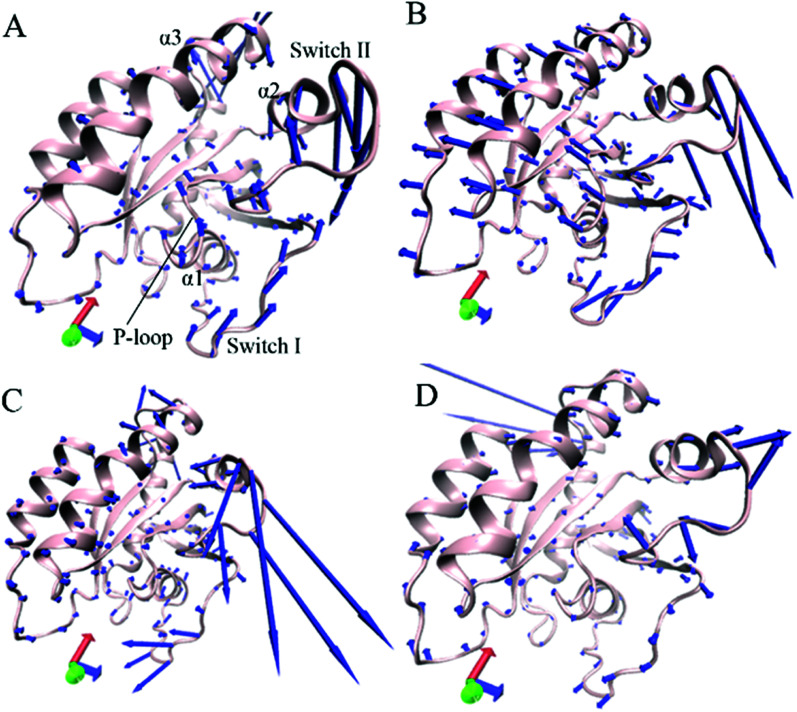
Concerted motions of structural domains in KRAS revealed by the first eigenvector from principal component analysis: (A) GTP-associated WT KRAS, (B) GTP-associated Q61A KRAS, (C) GTP-associated Q61H KRAS and (D) GTP-associated Q61L KRAS.

Through the above mentioned eigenvalues, the first eigenvector embodies main motion behavior of KRAS. Because of so, the first eigenvector from PCA is visually exhibited in Fig. 3 by aid of the software VMD and the optimized structure. It is found that the switch-I and switch-II show stronger movement by comparison with the other structural domain of KRAS. In the GTP-associated WT KRAS, two switches move toward a completely opposite direction and are close to each other, which results in a more compact switch domain, furthermore the switch-II displays the strongest movement among the structural domain (Fig. 3A). Compared to the WT state of KRAS, Q61A not only obviously enhances the motions of the P-loop and switch-II but also induces a tendency of the P-loop, switch-I and switch-II to move outwards, which leads to a more incompact binding pocket of GTP (Fig. 3B). By referencing the WT KRAS, Q61H changes the motion direction of the switch-II and strengthens its motion intensity, moreover makes the switch-I move toward the left, which also causes an untighter binding pocket of GTP (Fig. 3C). In the case of the GTP-bound Q61L KRAS, two switches move toward an opposite direction and are far away from each other, which forms an completely opening tendency of the switch domain (Fig. 3D).

Based on the above analyses, Q61A, Q61H and Q61L not only alter motion modes of the switch-II relative to the P-loop and switch-I but also obviously change motion intensity and dynamics behavior of the switch domain of KRAS, which yields significant influences on binding activity of KRAS. The Pantsar's work verified that conformational changes of the switch domain heavily affect the activity of KRAS.^16^ The μs-scaling MD simulation from Sayyed-Ahmad *et al.* indicated that mutation-mediated differences in dynamics and interaction networks can disturb binding of KRAS to GEFs and GAPs or effectors,^101^ which basically agrees with our current findings.

### 3.3 Difference in free energy landscapes induced by mutations

**Fig. 4:**
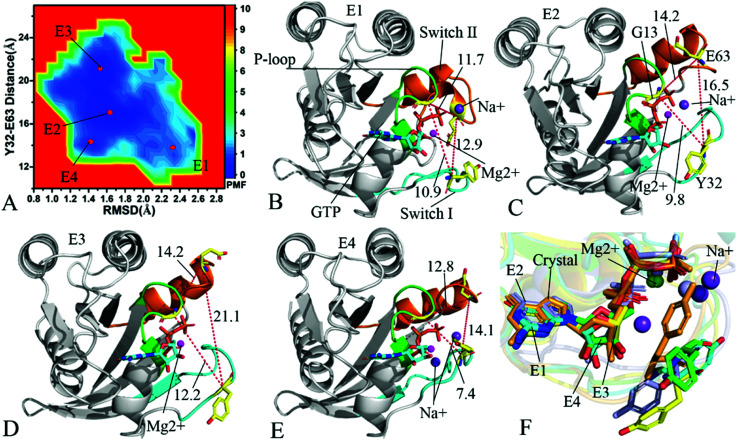
Free energy landscapes and structures of the GTP-bound Q61A KRAS: (A) showing free energy landscape constructed by using RMSD of backbone atoms and the distance of Y32 away from E63 as reaction coordinates, in which E1, E2, E3 and E4 indicate energy valleys detected by MR-GaMD simulations, (B), (C), (D) and (E) corresponding to representative structures located at energy valleys E1, E2, E3 and E4, respectively, and (F) displaying superimpositions of GTP, residue Y32 and ions in four representative structures with that in the crystal structure (6MNX). The unit of the distances is Å and that of free energy is kcal mol^−1^.

**Fig. 5:**
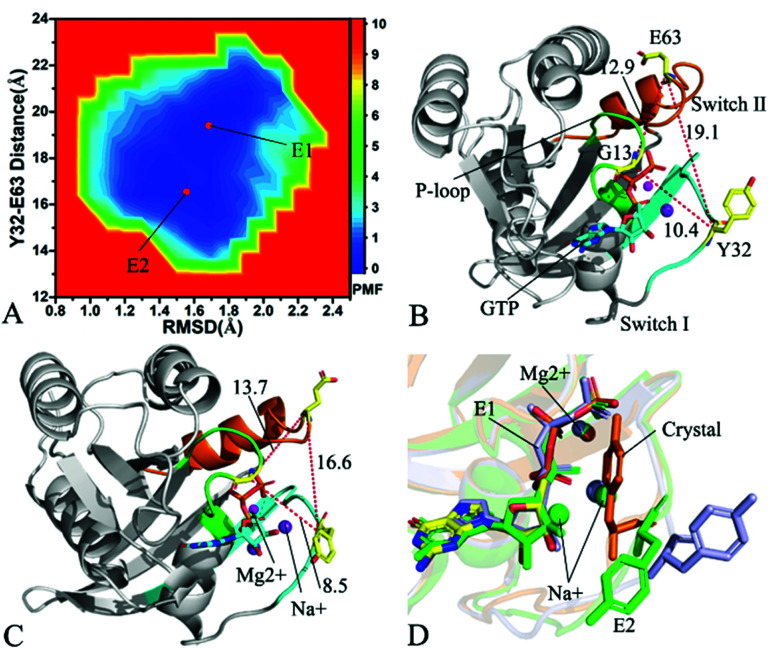
Free energy landscape and structures: (A) depicting free energy landscape built by utilizing RMSDs of backbone atoms and the distance of Y32 away from E63, (B) and (C) indicating the representative structures situated at energy valleys E1 and E2, and (D) suggesting superimposition of residue Y32, GTP and ions in two representative structures with that in the crystal structure (6MNX). The unit of the distances is Å and that of free energy is kcal mol^−1^.

**Fig. 6:**
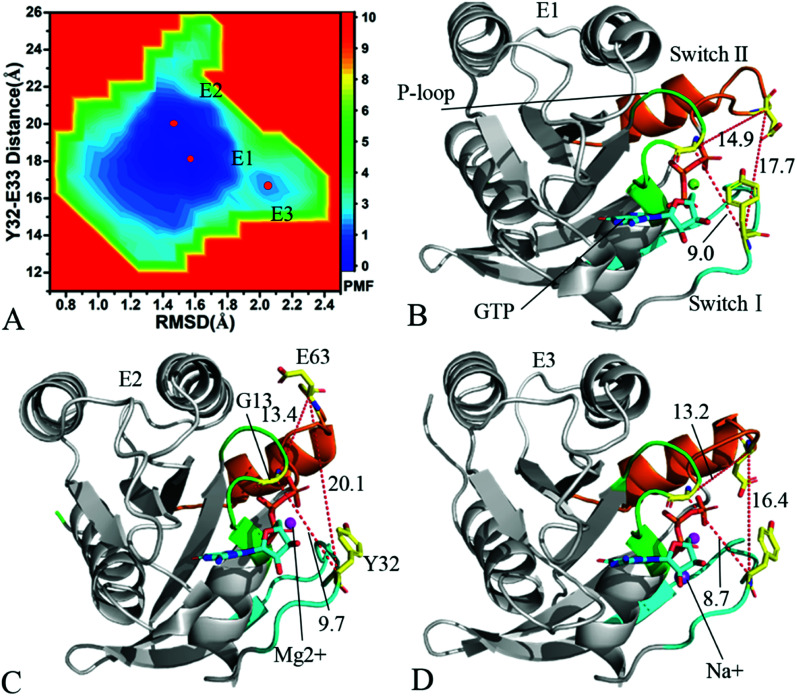
Free energy landscape and structures of the GTP-associated Q61L KRAS: (A) indicating free energy landscape built by employing RMSDs of backbone atoms and the distance of Y32 away from E63 as reaction coordinates, in which E1, E2 and E3 signify energy valleys identified by MR-GaMD simulations, (B), (C) and (D) displaying representative structures falling into energy valleys E1, E2 and E3, separately. The unit of the distances is Å and that of free energy is kcal mol^−1^.

To understand changes in free energies of KRAS induced by Q61 mutants, the RMSDs of backbone atoms and the distance of Y32 away from E63 were used as reaction coordinates to construct FELs of KRAS. RMSDs can efficiently reflect structural fluctuation of KRAS through the entire MR-GaMD simulation. Residues Y32 and E63 are situated at the switch-I and switch-II, respectively, and the alterations in the distance between them can rationally embody conformational transformation of the switch domain of KRAS. Therefore, we selected RMSDs and the distance between Y32 and E63 as reaction coordinates to build FELs. The constructed FELs and the extracted structural information from MR-GaMD simulations were displayed at Fig. 4–6 and S3–S6.†

For the GTP-bound WT KRAS, only an energy valley (E1) is detected by MR-GaMD simulations (Fig. S3A†). This result indicates that the conformations of the GTP-bound WT KRAS are mostly populated at an energetic space. The distance of Y32 away from E63 in the crystal structure (6MNX) is 19.3 Å and that in the structure E1 is ∼18.8 Å, moreover the distances of G13 away from E63 and T35 shows a small change between the crystal structure and the structure E1 (Fig. S3B and C†). The alignment between these two structures shows that the most regions of KRAS, GTP, magnesium ion Mg^2+^ and an important residue Y32 are stable in the GTP-bound WT state, only the switch-II has an obvious deviation.

In the case of the GTP-bound Q61A KRAS, MR-GaMD simulations capture four different energy valleys E1, E2, E3 and E4 (Fig. 4A), indicating that the conformations of the GTP-bound Q61A KRAS are mainly distributed at four energetic spaces. The distances of Y32 away from E63 in four energetic states E1, E2, E3 and E4 are 12.9, 16.5, 21.1 and 14.1 Å (Fig. 4B–E), respectively, which suggests that the switch domain of KRAS in energetic state E1 is the tightest while that of KRAS in energetic state E3 is the most incompact. Meanwhile the distances of G13 away from E63 and Y32 in four different energetic states also yield different changes, which also reflects alterations in sizes of binding pockets. Superimposition of four representative structures located at energy valleys E1–E4 with the crystal structure (6MNX) exhibits that the switch-I and switch-II are highly disordered (Fig. S4†), implying that Q61A produces significant effect on structural flexibility of the switch domain. More interestingly, a sodium ion Na^+^ appears at the structures E1 and E2 and two sodium ions Na^+^ are detected in the structure E4, which possibly changes electrostatic environment around the binding pocket. Superimposition of GTP, residue Y32 and ions in four representative structures with that in the crystal structure (6MNX) is depicted in Fig. 4F. The result suggests that Q61A hardly affects stability of GTP and Mg^2+^ through the entire MR-GaMD simulations but evidently alters conformations of Y32 in four energetic states by comparison with the crystal structure. Besides, Fig. 4F also shows that sodium ions (Na^+^) appear at different sites near the phosphate group of GTP and change electrostatic environment around GTP, but these sodium ions are unstable. The work of Xu *et al.* also indicated that conformational arrangement of the switch domain induced by Q61A also disturbs binding of KRAS to effectors, which is in good agreement with our current findings.^10^

With regard to the GTP-bound Q61H KRAS, MR-GaMD simulations identify two energetic valleys E1 and E2 and this result signifies that the conformations of the GTP-bound Q61H KRAS are primarily populated at two energetic spaces (Fig. 5A). In the energetic state E1, although the distance between Y32 and E63 of the Q61H KRAS is 19.1 Å and similar to that of the crystal structure (6MNX), but the distances of G13 away from E63 and Y32 in the Q61H KRAS have obvious difference compared to that in the crystal structure (Fig. 5B and S4B†). The distance of Y32 away from E63 in the energetic state E2 is ∼16.6 Å, which results in a more compact switch domain by referencing the GTP-bound WT KRAS (Fig. 5C). Alignment of the structures E1 and E2 with the crystal structure exhibits that Q61H generates evident influences on conformational alterations of the switch domain, especially for the switch-II (Fig. S5†). According to superimposition of GTP, ions and Y32 in the structures E1 and E2 of the GTP-bound Q61H KRAS with that in the WT one (Fig. 5D), Q61H hardly disturbs stability of GTP and Mg^2+^ but obviously alters conformations of Y32. Besides although sodium ions (Na^+^) appear near the phosphate group of GTP in the Q61H KRAS, these sodium ions have different sites in two structures E1 and E2 (Fig. 5D), hence these sodium ions certainly change electrostatic environment around GTP. Compared to the GTP-bound Q61A KRAS, the GTP-associated Q61H one has more stable conformations. The study from Zhou *et al.* unveiled that a hyperdynamic switch-II allows for a more stable interaction with the switch-I in the GTP-associated Q61H KRAS and their results also verified that an enhanced RAF activity stems from not only absent intrinsic GTP hydrolysis activity but also increased affinity for RAF.^51^ MD simulations performed by Lu *et al.* suggested that conformational transformation of the switch domain because of Q61H affects intrinsic GTPase activity.^34^ The Vatansever's work found that Q61H leads to distinctly strong deviations in the switch-II and decreases the number of conformations of the switch region in the inactive KRAS.^40^ These previous findings basically support our current work.

As for the GTP-bound Q61L KRAS, three energetic valleys E1, E2 and E3 are recognized by MR-GaMD simulations (Fig. 6A), demonstrating that conformations of the GTP-bound Q61L KRAS are primarily distributed at three energetic spaces. As shown in Fig. 6B–D, the distances between Y32 and E63 in three structures E1, E2 and E3 are 17.7, 20.1 and 16.4 Å, separately, thus the structures E1 and E3 form a tighter switch domain while the structure E2 has a more incompact one compared to the WT KRAS. Three structures E1, E2 and E3 are aligned together with crystal structure (Fig. S6A†) and the result suggests that Q61L exerts an apparent effect on the conformation of the switch domain of KRAS but barely affects stability of GTP and Mg^2+^. Meanwhile a sodium Na+ appears at the structure E3 (Fig. 6D), which possibly brings an impact on electrostatic environment near GTP. Superimposition of GTP, ions and Y32 in the structures E1–E3 with that in crystal structure verifies that conformations of Y32 are heavily affected by Q61L but GTP and Mg^2+^ are highly stable through the entire MR-GaMD simulations (Fig. S6B†). The crystal structure of the GTP-bound Q61L KRAS determined by Hunter *et al.* suggested that the switch-I and switch-II are disordered and KRAS exhibits a low level of intrinsic activity,^79^ which agrees well with the information revealed by our current study.

The aforementioned details reveal that Q61A, Q61H and Q61L highly affect structural stability of the switch domain and make the switch domain locate at a highly disordered state. In fact, the switch-I and switch-II take part in functional interactions of KRAS with GEFs and GAPs or effectors,^21^ hence high disorder of the switch domain of course affects the activity of KRAS. Multiple previous studies uncovered that conformational changes of the switch domain induced by mutations produce key influences on the intrinsic GTPase activity,^53,102–104^ which is in basic consistence with our results. Our work also finds that Q61A, Q61H and Q61L yield significant effect on conformations of Y32, which tunes the intrinsic activity of KRAS. It has also been reported that residue Y32 situated at the switch-I plays a significant role in intrinsic hydrolysis of KRAS,^26,79,105,106^ thus changes in the orientation of the sidechain in Y32 directly disturb the activity of KRAS. More interestingly, our work verifies that Q61A, Q61H and Q61L induces a possibility of sodium ions (Na^+^) appearing in different energetic structures, which possibly yields an important impact on electrostatic environment around binding sites. This interesting phenomenon is also observed at the previous studies,^30,46^ supporting our current findings.

### 3.4 Alterations in interaction network of GTP with KRAS caused by Q61 mutants

**Fig. 7:**
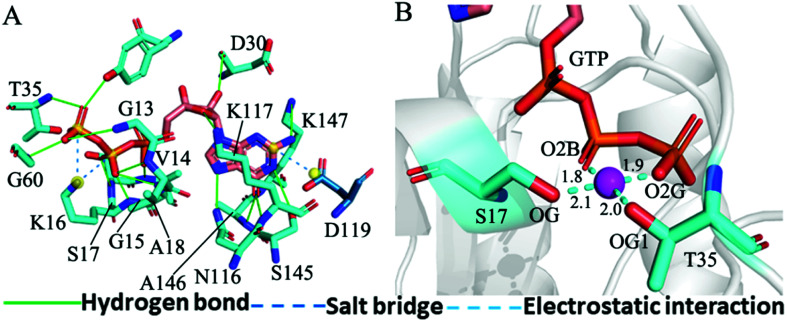
Interaction network in the GTP-bound WT KRAS: (A) interactions of GTP with key residues, including hydrogen bonds and salt bridge and (B) electrostatic interactions of magnesium ion Mg^2+^ with residues and GTP. The unit of the distances is Å.

**Fig. 8:**
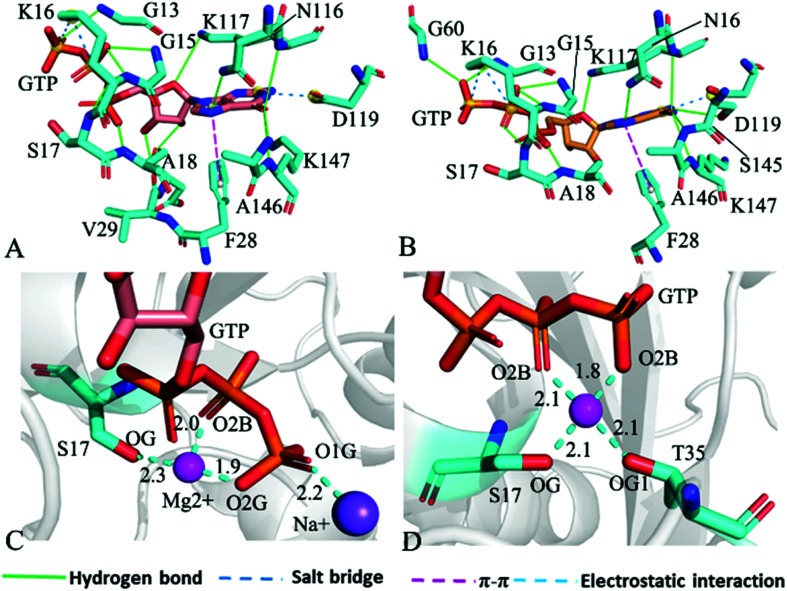
Interaction network in the GTP-bound Q61A KRAS: (A) interactions of GTP with residues in compact state of the switch domain, (B) interactions between GTP and residues in uncompact state of the switch domain, (C) interactions of magnesium ion Mg^2+^ and sodium ion Na^+^ with GTP and residues and (D) interactions of magnesium ion Mg^2+^ with GTP and residues.

Identification of ligand–receptor interaction network is highly helpful for understanding molecular mechanism on binding of ligands to receptors. The PLIP sever was applied to analyze the interaction network of GTP with KRAS and the results were displayed at Fig. 7, 8, S7 and S8.† HBIs of GTP with KRAS were also explored by using the CPPTRAJ module in Amber 20 and the information of HBIs were given in Table 1. HBIs are determined through a standard of an acceptor⋯donor distance of <3.5 Å and acceptor⋯H-donor angle of >120°. The occupancy of hydrogen bonds listed in Table 1 reflects stability of HBIs though the entire MR-GaMD simulations.

**Table 1:** Hydrogen bonding interactions of GTP with the WT and mutated KRAS

Hydrogen bonds	aOccupancy (%)				
Residue	GTP	WT	Q61A	Q61H	Q61L
G13–N–H	O3B	93.1	44.2	51.2	91.3
V14–N–H	O1B	25.9	28.2	25.1	26.3
G15–N–H	O1B	98.1	98.3	97.8	97.9
K16–N–H	O1B	99.9	99.9	99.9	99.9
S17–N–H	O2B	99.6	98.7	99.6	99.6
A18–N–H	O1A	99.7	99.6	99.6	99.7
V29–N–H	O2′-HO′2	65.8	45.3	40.1	36.8
D30–O	O3′-H3T	54.2	30.2	34.1	33.6
T35–N–H	O1G	3.5	4.2	29.2	15.3
G60–N–H	O3G	92.1	56.7	78.7	72.7
N116–ND2–HD2	N7	82.6	83.1	80.8	80.6
K117–N–H	O4′	40.1	45.4	44.8	44.7
S145–OG–HG	N1	66.9	66.2	37.8	43.4
A146–N–H	O6	75.6	72.3	73.0	76.6
K147–N–H	O6	73.1	74.1	75.3	71.0

a Occupancy (%) is defined as the percentage of simulation time that a specific hydrogen bond exists.

The structure E1 extracted from the single integrated MR-GaMD trajectory of the GTP-bound WT KRAS was used to analyze interaction network with the PLLP sever (Fig. 7). It is found from Fig. 7A that GTP yields HBIs with six residues G13, V14, G15, K16, S17 and A18 situated at the P-loop and except for V14, the occupancy of HBIs are higher than 93.1% (Table 1), indicating that HBIs of GTP with G13, G15, K16, S17 and A18 are extremely stable. According to Table 1, GTP forms three hydrogen bonds with residues V29, D30 and T35 located at the switch-I and their occupancy is lower than 65.8% and the hydrogen bond between GTP and V29 does not appear at the structure E1 (Fig. 7A). It is also observed that GTP produces a HBI with residue G60 in the switch-II and its occupancy is 92.1%, implying that this hydrogen bond is highly stable through the entire MR-GaMD simulations in the GTP-bound WT state of KRAS (Table 1 and Fig. 7A). In addition, GTP generate HBIs with residues N116, S145, A146 and K147 with the occupancy higher than 66.9% (Table 1 and Fig. 7A), showing that these hydrogen bonds have a higher stability. Although GTP also forms a hydrogen bond with K117, its occupancy is only 21.1%, hence this hydrogen bond shows a weak stability during MR-GaMD simulations. Based on Fig. 7A, the phosphate group of GTP produces two salt bridge interactions with K16 and the adenine group of GTP forms a salt bridge interaction with D119. These three salt bridges play a significant role in stabilization of binding of GTP to KRAS. Meanwhile, the PLIP server also detects electrostatic interactions of magnesium ion Mg^2+^ with GTP, residues S17 and T35 (Fig. 7B), thus the appearance of Mg^2+^ between GTP and S17 is helpful for maintaining stability of the P-loop conformation.

**Fig. 9:**
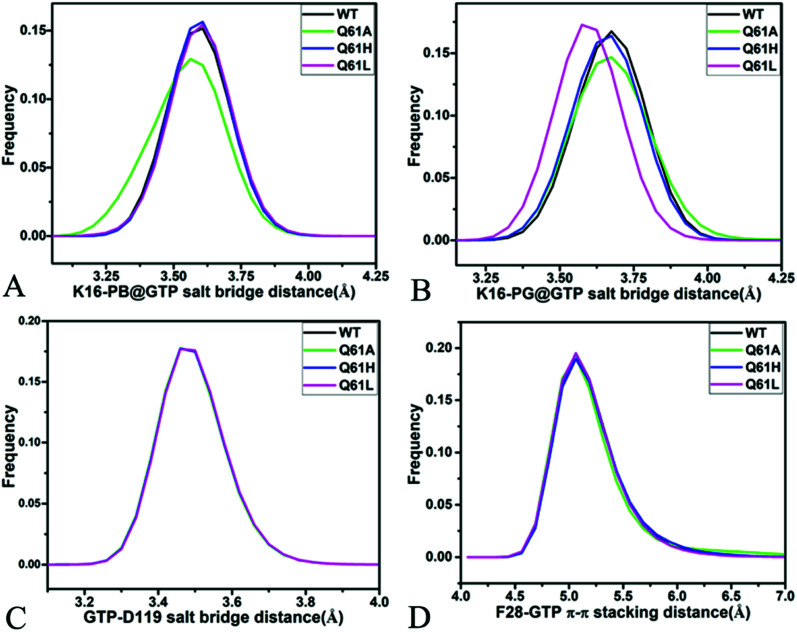
Key interactions between GTP and several residues in the WT and mutated KRAS: (A) salt bridge interaction between K16 and phosphorus atom PB of GTP, (B) salt bridge interactions of K16 with phosphorus atom PG of GTP, (C) salt bridge interaction between D119 and GTP and (D) π–π stacking interaction of F28 with GTP.

For the GTP-bound Q61A, Q61H and Q61L KRAS, the most compact and incompact structures taken from the single integrated MR-GaMD trajectory were adopted to analyze the interaction network of GTP with three mutated KRAS using the PLIP server (Fig. 8, S7 and S8†). It is noted that Q61A, Q61H and Q61L KRAS share almost same binding spots of GTP as the WT KRAS. As a common feature, three salt bridge interactions of GTP with K16 and D119 can be identified in all energetic states of the GTP-bound WT and mutated KRAS (Fig. 7A, 8A, B, S7A, B, S8A and B†). To understand effect of Q61A, Q61H and Q61L on these salt bridge interactions, the distances of the positive charge in K16 away from the phosphorus atoms PB and PG of GTP and that of the negative charge in D119 away from the adenine group of GTP were computed by means of the single integrated MR-GaMD trajectory (Fig. 9A–C). The results exhibit that Q61A, Q61H and Q61L hardly produce obvious influences on the salt bridge between D119 and the adenine group of GTP (Fig. 9C), but Q61A slightly strengthens the salt bridge interaction between K16 and the phosphorus atom PB of GTP compared to three other states and Q61L enhances the salt bridge interaction between K16 and the phosphorus atom PG of GTP by referencing three other states (Fig. 9A and B). A π–π interaction between the phenyl group of F28 and the adenine group of GTP was recognized in the compact and incompact states of the GTP-associated Q61A KRAS (Fig. 8A and B) ant the incompact states of the GTP-bound Q61H and Q61L KRAS (Fig. S7B and S8B†). To understand stability of this π–π interaction at four simulated systems, the distance between the phenyl group of F28 and the adenine group of GTP was estimated and the results were depicted in Fig. 9D. As a result, the distance of this π–π interaction is distributed at 5.1 Å in four simulated systems and the currently studied Q61 mutants hardly generate evident impacts on stability of this π–π interaction. Although the π–π interaction between F28 and GTP does not appear at the structure E1 extracted from MR-GaMD simulations of the GTP-bound WT KRAS (Fig. 7A), this π–π interaction is still stably maintained through MR-GaMD simulation (Fig. 9D). In addition, a cation–π interaction of K117 with the adenine of GTP is recognized at the incompact state of the GTP-bound Q61H and Q61L KRAS (Fig. S7B and S8B†).

**Fig. 10:**
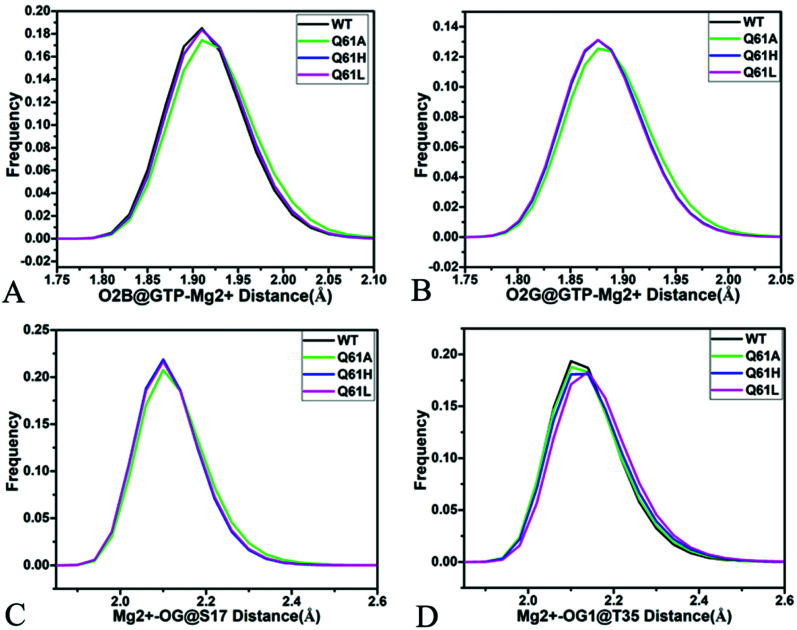
Interactions of magnesium ion Mg^2+^ with GTP and residue: (A) interaction between Mg^2+^ and oxygen atom O2B of GTP, (B) interaction of Mg^2+^ with oxygen atom O2G of GTP, (C) interaction between Mg^2+^ and oxygen atom OG of S17 and (D) interaction of Mg^2+^ with oxygen atom OG1 of GTP.

As shown in Fig. 7B, 8C, D, S7C, D, S8C and D,† the PLIP server also detects electrostatic interactions of magnesium ion Mg^2+^ with the oxygen atoms OG of S17, OG1 of T35, O2B and O2G of GTP, but the interaction of Mg^2+^ with T35 does not appear in the compact state of the GTP-bound Q61A KRAS (Fig. 8C). The distances of these oxygen atoms away from Mg^2+^ were calculated and their frequency distribution were plotted at Fig. 10. The information demonstrates that Q61A, Q61H and Q61L do not affect stability of Mg^2+^ through the entire MR-GaMD simulations. Thus electrostatic interactions of Mg^2+^ with GTP and S17 is helpful for stabilizing the interaction of GTP with the P-loop. Besides, sodium ions Na+ appear near the phosphate group of GTP at different states of the GTP-associated Q61A, Q61H and Q61L KRAS (Fig. 8C, S7C, D and S8C†), and these sodium ions (Na^+^) interact with different oxygen atoms in the phosphate group of GTP, which changes electrostatic environment around GTP and affects conformational changes of KRAS.

The distinct difference in interaction network of GTP with the WT, Q61A, Q61H and Q61L KRAS primarily stems from HBIs of GTP with residues V29 and D30 in the switch-I. Compared to the GTP-associated WT KRAS, the occupancy of the hydrogen bond between GTP and V29 is decreased by 20.5, 25.8 and 29.0% because of Q61A, Q61H and Q61L, respectively, while that of the hydrogen bond between GTP and D30 is reduced by 24.0, 20.1 and 20.6%, separately (Table 1). Therefore the decrease in stability of hydrogen bonds between GTP and residues V29 and D30 is responsible for the high disorder of the switch-I observed from the previous FEL analyses. The occupancy of the hydrogen bond between GTP and G60 is reduced by 35.4, 13.4 and 19.4% due to Q61A, Q61H and Q61L by comparison with the WT KRAS, respectively, which plays a key role in the high disorder of the switch-II seen from the previous FEL analyses (Table 1). Apart from effect of Q61A and Q61H on the HBI of GTP with G13, three Q61 mutants hardly disturb stability of HBIs of GTP with V14, G15, K16, S17 and A18 in the P-loop (Table 1), which rationally explains the high order of the P-loop observed from previous structural superimposition. In addition, Q61A, Q61H and Q61L do not yield evident impacts on stability of HBIs of GTP with N116, K117, S145, A146 and K147 located at the non-switch regions compared to the WT KRAS (Table 1) and this result verifies a key factor maintaining structural order of the non-switch domain.

According to the aforementioned results, the following conclusions can be drawn: (1) salt bridge interactions of K116 and D119 with GTP and electrostatic interactions of Mg^2+^ with GTP, S17 and T35 play a key role in maintaining stability of the GTP-KRAS binding, (2) the decrease in stability of HBIs of GTP with V29 and D30 induced by Q61A, Q61H and Q61L is responsible for structural disorder of the switch-I and that in the occupancy of the hydrogen bond between GTP and G60 leads to structural disorder of the switch-II, and (3) the appearance of sodium ions near the phosphate group of GTP in the mutated KRAS alters electrostatic environment around GTP and affects the activity of KRAS.

## 4 Conclusions

KRAS has been regarded as an important drug target for cancer treatment and its conformational changes caused by ligand bindings and mutations are requisite for drug design. In this work, 4.8-μs MR-GaMD simulations were performed on the GTP-bound WT, Q61A, Q61H and Q61L KRAS to explore effect of Q61 mutants on the activity of KRAS. The calculated RMSDs of backbone atoms show that Q61A, Q61H and Q61L enhance total structural fluctuations of KRAS and the estimated RMSF difference of the *C*_α_ atoms between the WT and mutated KRAS suggest that Q61A, Q61H and Q61L obviously alter structural flexibility of the switch domain in KRAS, which affects the activity of KRAS. The information stemming from cross-correlation analysis and PCA verifies that three Q61 mutants affect motion modes of the switch domain and evidently change dynamics behavior of the switch domain in KRAS, which in turn regulates binding of KRAS to effectors.

FELs were constructed by utilizing RMSDs of backbone atoms and the distance of Y32 away from E63 as reaction coordinates. The results display that Q61A, Q61H and Q61L lead to high disorder of the switch domain in KRAS and reveal energetic basis with regard to conformational changes of KRAS. Moreover, MR-GaMD simulations detect the appearance of sodium ions (Na^+^) around the phosphate group of GTP in different structures falling into energy valleys, which changes electrostatic environment around GTP and certainly affects conformational changes of KRAS.

The analysis of interaction network reveals that the decrease in stability of HBIs of GTP with the switch-I and switch-II is responsible for the structural disorder of the switch domain. Dynamic analysis suggests that the flexibility change of the loop L2 is involved in the regulation on allosteric sites of KRAS and affects the alteration in allosteric binding pocket, which provides a hint for design and development of inhibitors tuning the activity of KRAS in the future.

## Conflicts of interest

The authors declare that they have no known competing financial interests.

## Supplementary Material

RA-012-D1RA07936K-s001
