# Supplementary material for: Q61 mutant-mediated dynamics changes of the GTP-KRAS complex probed by Gaussian accelerated molecular dynamics and free energy landscapes
Source: RSC Adv. 2022 Jan 11;12(3):1742–57. doi: 10.1039/d1ra07936k (PMC8978876; doi:10.1039/d1ra07936k)
Supplement: RA-012-D1RA07936K-s001 [file RA-012-D1RA07936K-s001.pdf]

Electronic Supplementary Material (ESI) for RSC Advances.
This journal is © The Royal Society of Chemistry 2022

Q61 mutant-mediated dynamics changes of GTP-KRAS compound probed by Gaussian accelerated molecular dynamics and free energy landscapes†

Jianzhong Chen[a,*], Qingkai Zeng[a], Wei Wang[a], Qingquan Hu[a] and Huayin Bao[b,*]

* Corresponding author.
[a]*School of Science, Shandong Jiaotong University, Jinan, 250357, China E-mail: chenjianzhong1970@163.com, jzchen@sdjtu.edu.cn (J. Chen),*
[b]*School of Pharmacy, Shandong University of Traditional Chinese Medicine, Jinan, 250355, China.*
E-mail: baohuayin@163.com (H. Bao).

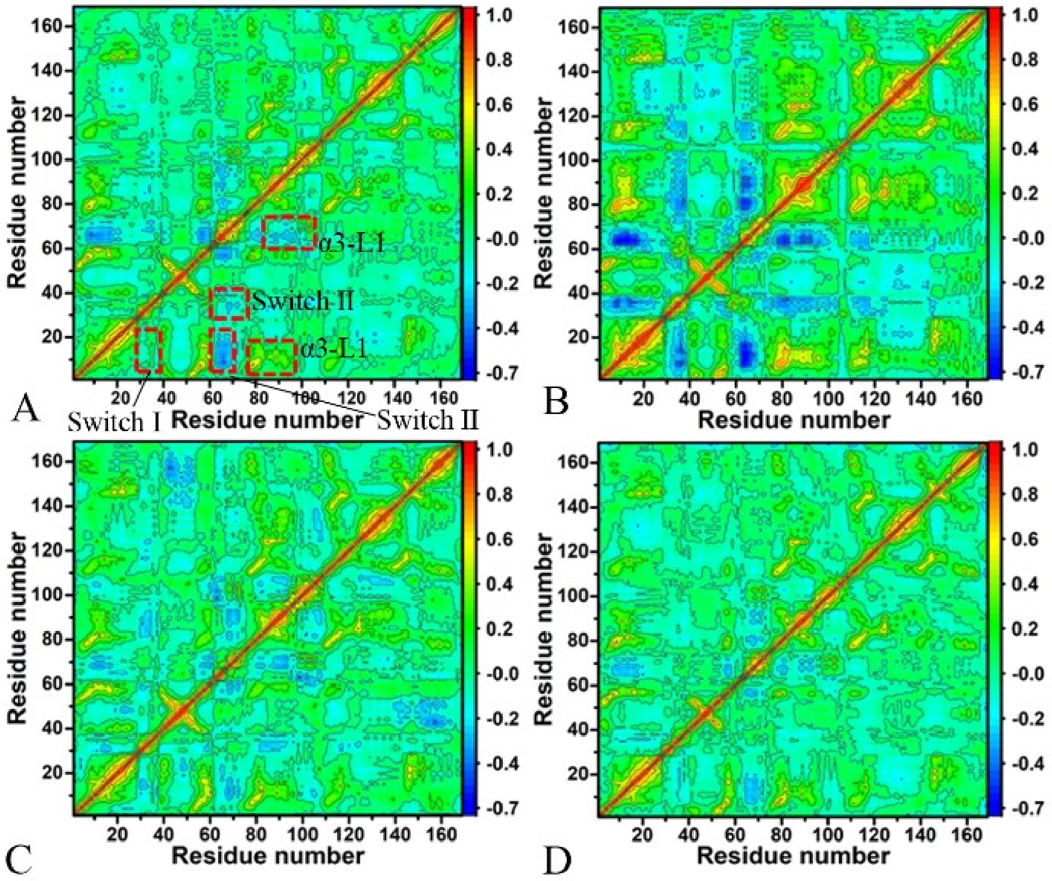

Fig. S1 Cross-correlation matrix calculated by using the coordinates of the C_α atoms recorded in the single integrated MR-GaMD trajectory: (A) GTP-bound WT KRAS, (B) GTP-bound Q61A KRAS, (C) GTP-bound Q61H KRAS and (D) GTP-bound Q61L KRAS.

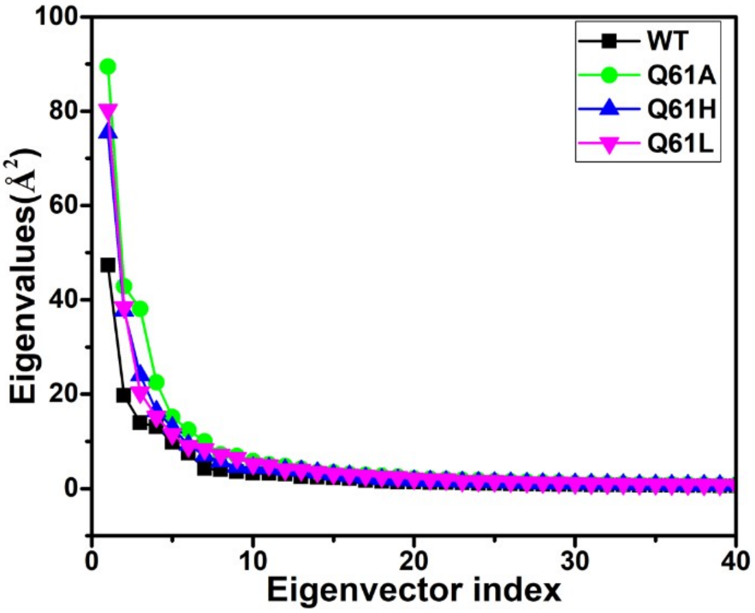

Fig. S2 Function of eigenvalues vs eigenvector indexes from principal component analysis, which is used to describe motion intensity of KRAS.

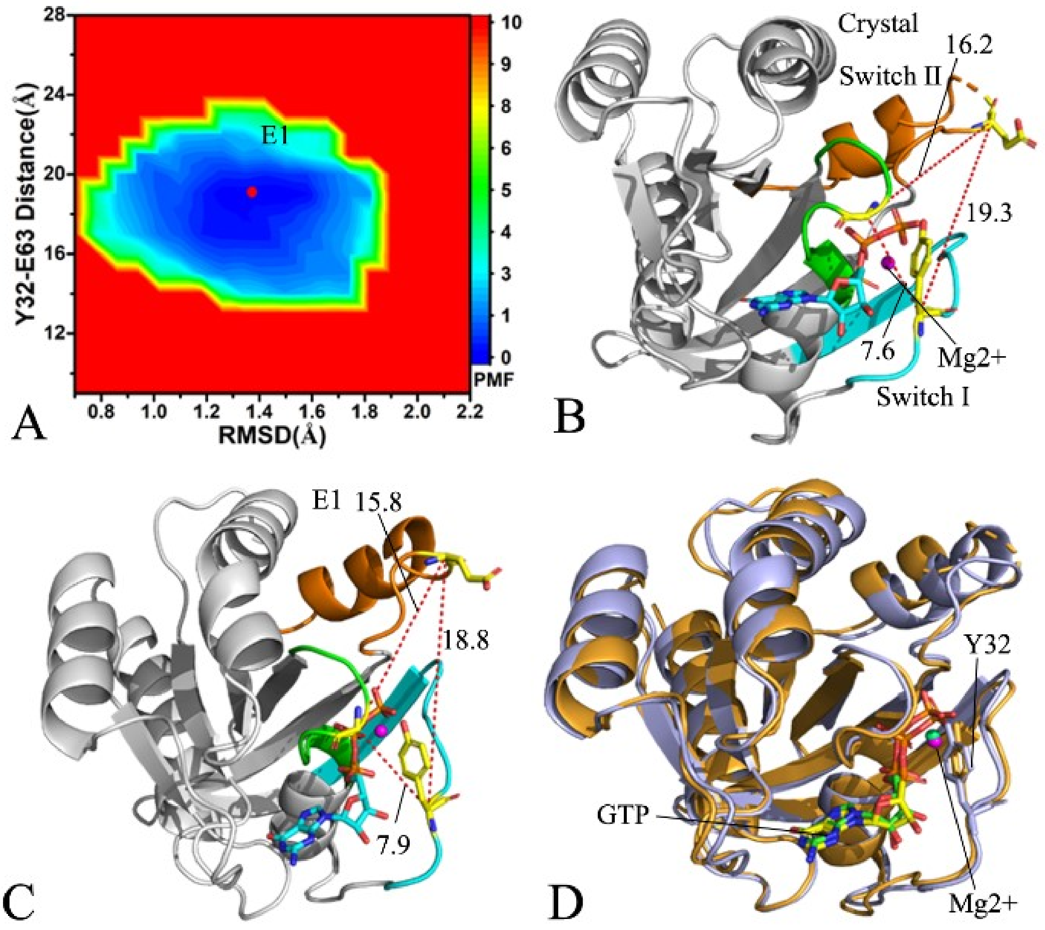

Fig. S3 Free energy landscapes and structures: (A) free energy landscape identified by MR-GaMD simulations, (B) crystal structure of the GTP-bound KRAS (PDB ID: 6MNX), (C) simulated structure located at energy valley E1 and (D) superimposition of crystal structure and simulated structure. The unit of all distances is Å and that of free energy is kcal/mol.

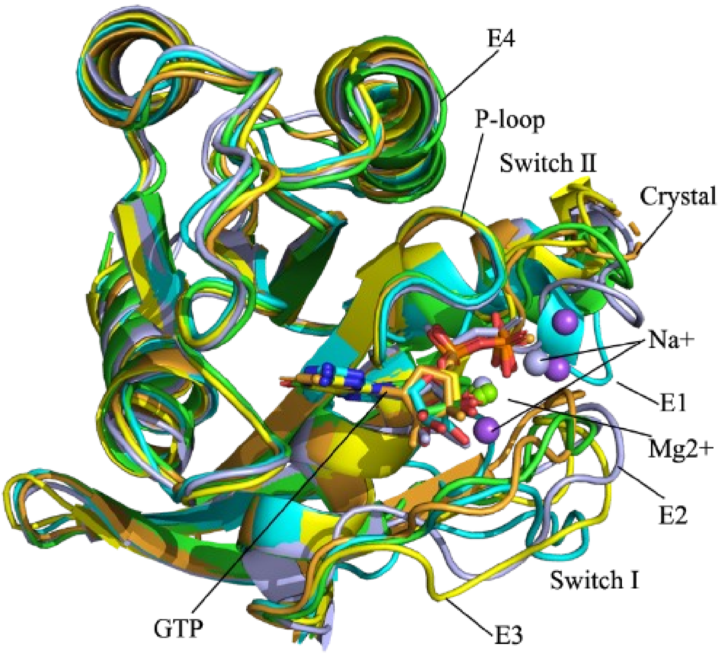

Fig. S4 Superimpositions of four representative structures of the GTP-bound Q61A KRAS situated at energy valleys E1, E2, E3 and E4 with crystal structure 6MNX.

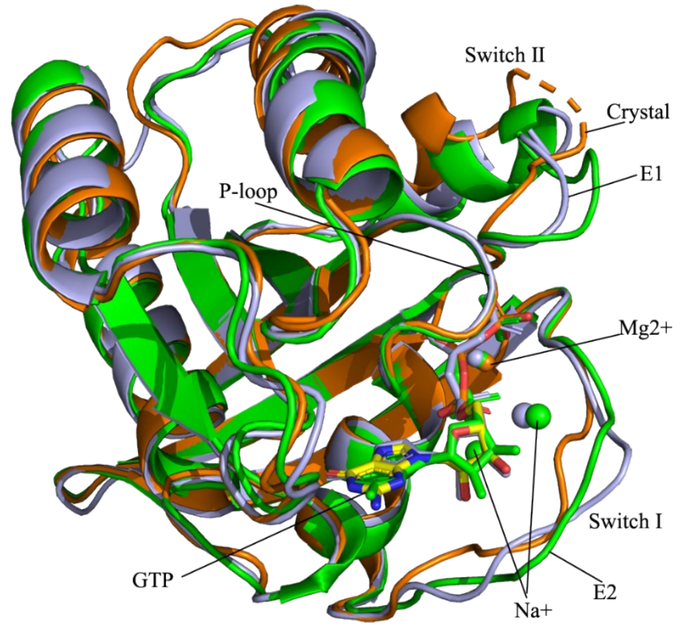

Fig. S5 Superimpositions of four representative structures of the GTP-bound Q61H KRAS situated at energy valleys E1 and E2 with crystal structure 6MNX.

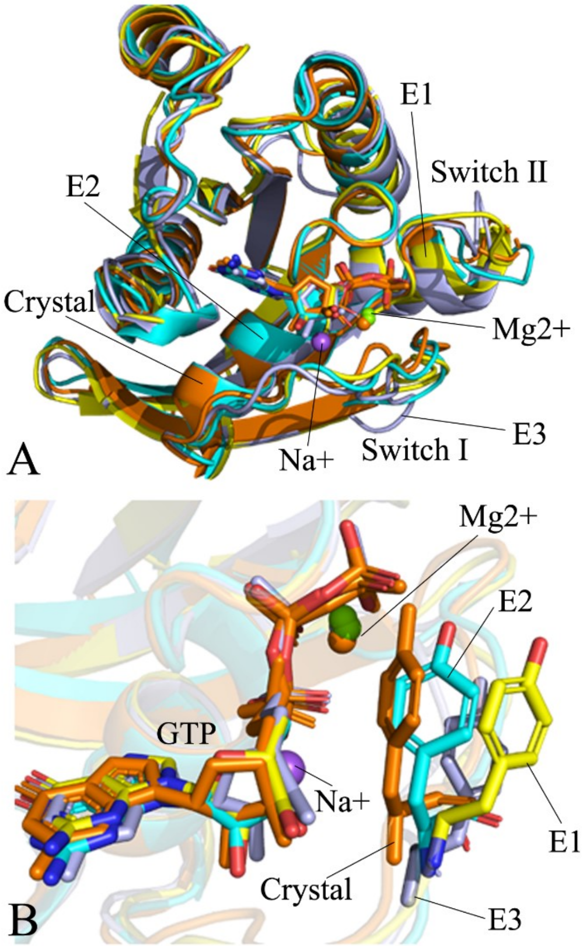

Fig. S6 (A) superimposition of three structures E1, E2 and E3 of the GTP-bound Q61L KRAS with the crystal structure (6MNX) and (B) alignment of GTP, ions and Y32 in three structures E1, E2 and E3 of the GTP-associated Q61L KRAS with that in the crystal structure (6MNX).

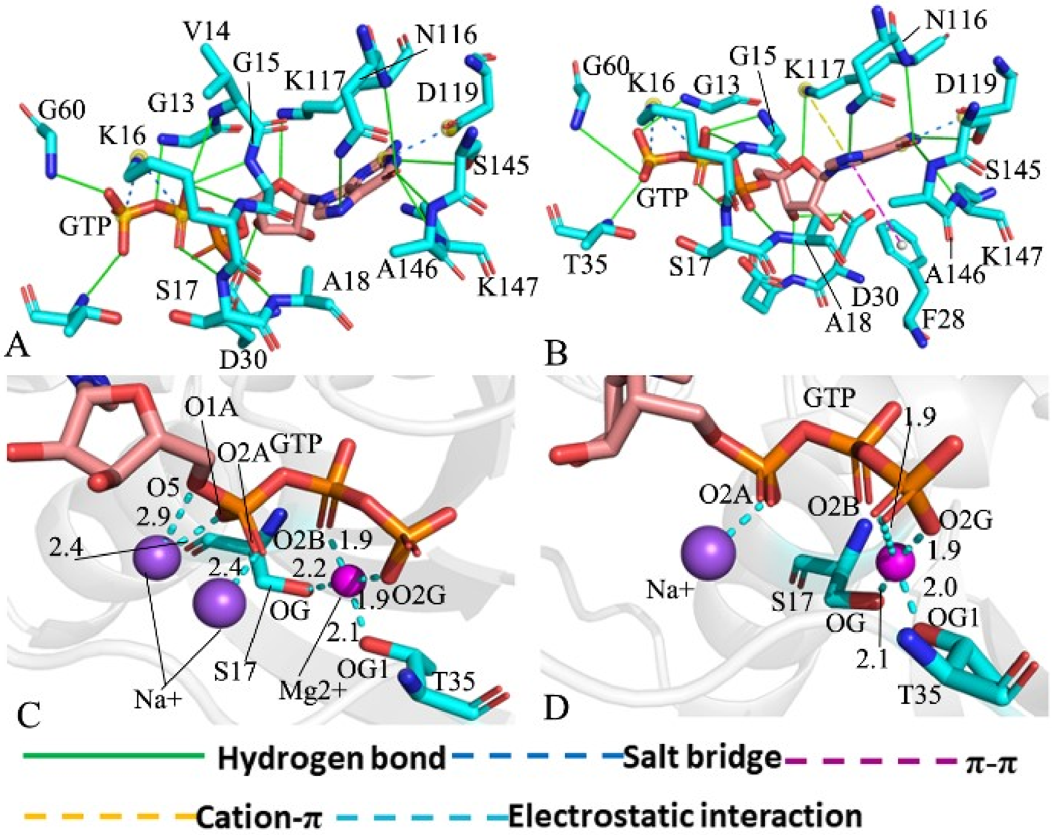

Fig. S7 Interactions of key residues in the GTP-bound Q61H KRAS: (A) interactions of GTP with residues in compact state of the switch domain, (B) interactions between GTP and residues in uncompact state of the switch domain, (C) interactions of magnesium ion Mg2+ and sodium ions Na+ with GTP and residues and (D) interactions of magnesium ion Mg2+ and sodium ion Na+ with GTP and residues.

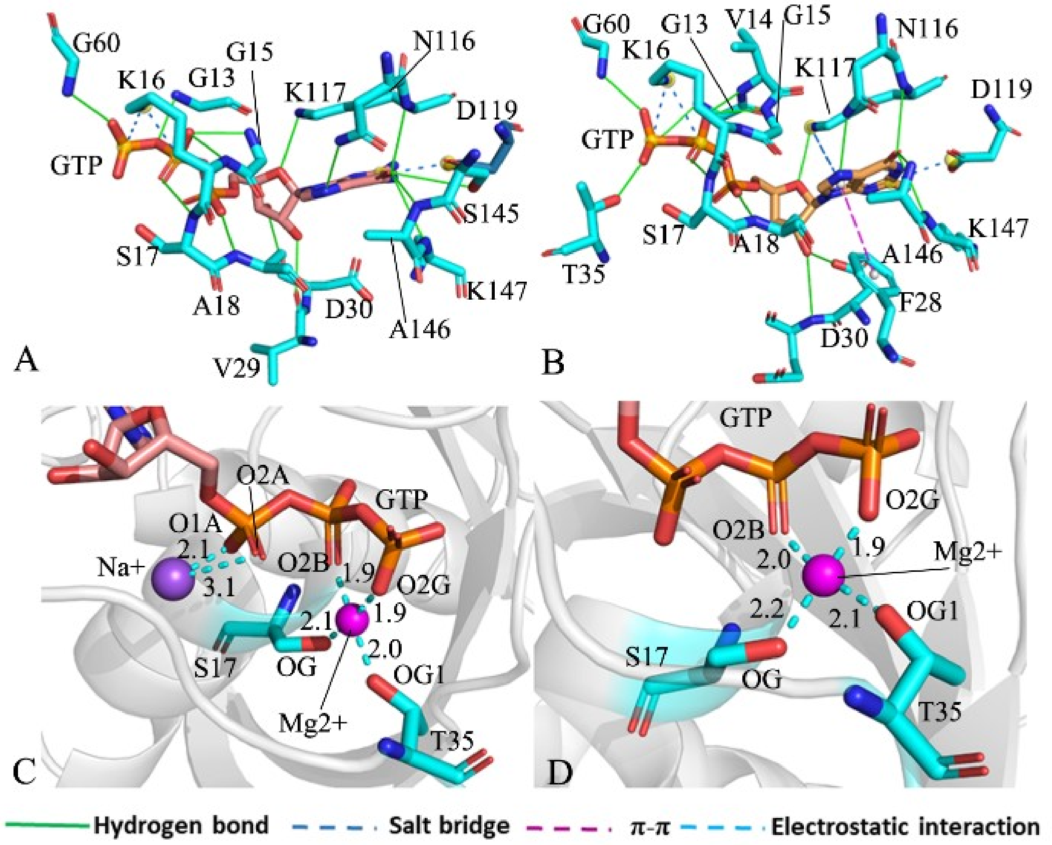

Fig. S8 Interactions of key residues in the GTP-bound Q61L KRAS: (A) interactions of GTP with residues in compact state of the switch domain, (B) interactions between GTP and residues in uncompact state of the switch domain, (C) interactions of magnesium ion Mg2+ and sodium ion Na+ with GTP and residues and (D) interactions of magnesium ion Mg2+ with GTP and residues.
